# A *De Novo* Genome Assembly Algorithm for Repeats and Nonrepeats

**DOI:** 10.1155/2014/736473

**Published:** 2014-05-25

**Authors:** Shuaibin Lian, Qingyan Li, Zhiming Dai, Qian Xiang, Xianhua Dai

**Affiliations:** ^1^School of Information Science and Technology, Sun Yat-Sen University, Guangzhou High Education Mega City, No. 132 Waihuan East Road, Panyu District, GuangZhou 510006, China; ^2^SYSU-CMU Shunde International Joint Research Institute, Shunde 528300, China

## Abstract

*Background.* Next generation sequencing platforms can generate shorter reads, deeper coverage, and higher throughput than those of the Sanger sequencing. These short reads may be assembled* de novo *before some specific genome analyses. Up to now, the performances of assembling repeats of these current assemblers are very poor.* Results.* To improve this problem, we proposed a new genome assembly algorithm, named SWA, which has four properties: (1) assembling repeats and nonrepeats; (2) adopting a new overlapping extension strategy to extend each seed; (3) adopting sliding window to filter out the sequencing bias; and (4) proposing a compensational mechanism for low coverage datasets. SWA was evaluated and validated in both simulations and real sequencing datasets. The accuracy of assembling repeats and estimating the copy numbers is up to 99% and 100%, respectively. Finally, the extensive comparisons with other eight leading assemblers show that SWA outperformed others in terms of completeness and correctness of assembling repeats and nonrepeats.* Conclusions.* This paper proposed a new* de novo *genome assembly method for resolving complex repeats. SWA not only can detect where repeats or nonrepeats are but also can assemble them completely from NGS data, especially for assembling repeats. This is the advantage over other assemblers.

## 1. Background

Over the past twenty years, genome sequencing technologies have made great progress in many aspects, such as speed, cost, coverage, and so forth. The automated Sanger sequencing is regarded as the first-generation genome sequencing technology which has the ability to read longer than 1000 base pair (1000–2000 bp). The latter sequencing technologies are referred to as the next-generation sequencing (NGS) technologies. Currently, the available commercial NGS platforms include GA, MiSeq, and HiSeq from Illumina [1], SOLiD and Ion Torrent from Life Technologies [2], RS system from Pacific Bioscience, and Heliscope from Helicos Biosciences [3–7]. Next generation sequencing machines can sequence the whole human genome in a few days, and this capability has inspired a flood of new projects that are aimed at sequencing large kinds of animals and plants [8, 9]. NGS can be characterized by highly parallel operation, higher yield, simpler operation, shorter reads, and much lower cost [10]. However, the NGS technologies all share a common intrinsic characteristic of providing very short read length (30~250 bp), which is substantially shorter than the Sanger sequencing reads.

These short reads may be assembled* de novo* before further genome analysis if the reference genome is not available. Currently, there are tens of genome assembly algorithms and software. Among them ABySS [11], ALLPATHS-LG [12], Bambus2 [13], CABOG [14], MSR-CA (http://www.genome.umd.edu/masurca.html), SGA [15], SOAPdenovo [16], and Velvet [17] are the typical ones. Each of them is able to run large and whole genome assembly using NGS short read data from mate-pair or paired-end information. In terms of repeats smaller than read length, these current assemblers performed well. But for the repeats longer than read length, most of them performed poorly in completeness and accuracy of assembling repeats from NGS data. It is shown that repetitive DNA comprises a significant fraction of the eukaryotic genomes, for example, ~20% of* Caenorhabditis elegans* and* Caenorhabditis briggsae* genomes [18] and ~50% of the human genome [19] have been identified as repetitive DNA. Most of these repetitive DNA sequences have some important biomedical functions and are closely related to some complex disease [20, 21], such as cancer [22], neuropsychiatric disorders [23], and autism [24]. Thus, it is necessary to improve the genome assembly algorithms, especially in assembling repeats.

To this end, we proposed a new genome assembly algorithm aiming for assembling repeats and nonrepeats, named SWA (sliding window assembly), which can assemble repeats and nonrepeats completely and accurately. In SWA, sliding window function is used to filter out the sequencing bias caused by sequencing process and improve the confidence of separating repeats and nonrepeats. There are five typical properties.

1. Assembling repeats and nonrepeats completely and accurately. SWA can assemble repeats and nonrepeats from NGS data directly in a parallel way which can reduce the memory usage and executive time. In addition, SWA cannot only detect where repeats or nonrepeats are but also can assemble them completely. Therefore, SWA provides an alternative solution to resolve long repeats in some extent.
2. Adopting dynamic overlapping strategy to extend each seed. The so-called dynamic overlapping strategy is to compute the reads overlapped with seed in intervals composed of maximum overlap and minimum overlap. This strategy can search the optimal read for extension in dynamic interval and jump over the short repeats.
3. Adopting sliding window to filter out the sequencing bias so as to improve the confidence of detecting boundary of repeats. NGS data is always full of sequencing bias and is highly uneven, which caused the difficulty of distinguishing where repeats or nonrepeats are. Sliding window technique is used to filter out the sequencing bias in the genome assembly process so as to increase the confidence of detecting boundary of repeats.
4. Proposing a compensational mechanism for the loss caused by low coverage. Low coverage makes the statistical properties of read counts less significant. To improve the statistical significance, SWA proposed a compensational mechanism based on sliding window. This mechanism can improve the statistical significance of read counts under the condition of low coverage.
5. Estimating copies of assembled ones as an auxiliary function.

The main contributions of our approach are as follows. (1) Assembling repeats and nonrepeats completely and accurately rather than only detecting where repeats or nonrepeats are. Complex repeats structures have very important biomedical functions. Consequently, the completeness and accuracy of assembling repeats are what SWA is mainly concerned about the completeness of assembled repeats and nonrepeats rather than the continuity of whole genome assembly. (2) Sliding window functions to filter out the sequencing bias are used in genome assembling process. Filtering noise by window function is very common in information processing but is rare in genome assembly process. SWA adopts sliding window to filter out NGS data bias and improve the statistical significance of read counts. In addition, a compensational mechanism based on sliding window was embedded in SWA. This mechanism can improve the significance of read counts under the condition of low coverage.

The assessments were performed in simulated datasets and real NGS datasets which are all generated by Illumina sequencer. Simulated study is only used to validate the performances of SWA. Therefore, it has little meaning to compare with other assemblers in simulated datasets. Extensive comparisons were conducted with other eight famous assemblers, such as ABySS, ALLPATHS-LG, Bambus2, CABOG, MSR-CA, SGA, SOAPdenovo, and Velvet, in real NGS datasets. The results indicate that for whatever small genomes or large genomes, SWA outperformed other eight leading assemblers in the completeness of assembling repeats. SWA is freely available at http://222.200.182.71/swa/SWA.rar.

## 2. Results

### 2.1. SWA Algorithm

**Figure 1:**
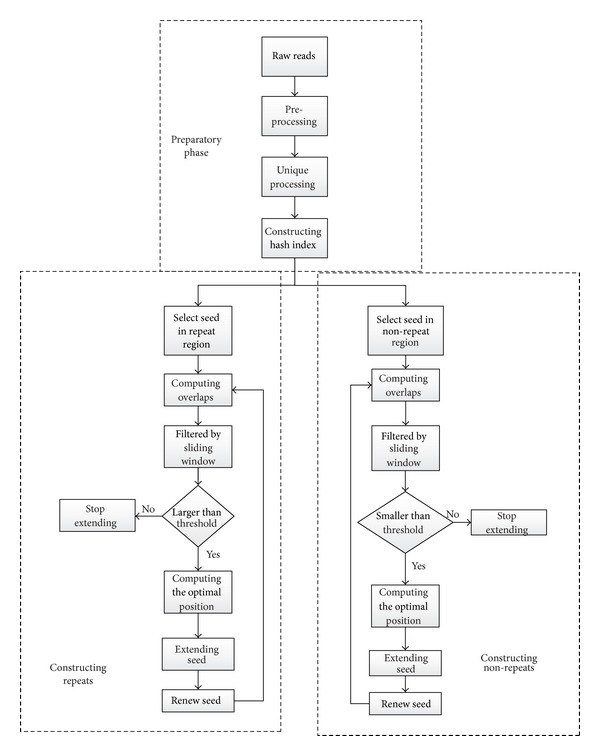
High-level diagram of the SWA assembly pipeline. The assembly has three main modules: preparatory stage, constructing repeats, and constructing nonrepeats. Preparatory stage includes data cleaning, unique processing, and hash index constructing. The stage of constructing repeats and constructing nonrepeats can be performed in parallel or in series.  Figure 1 shows the parallel manner. The specific steps of constructing repeats and nonrepeats are detailed in Methods.

**Figure 2:**
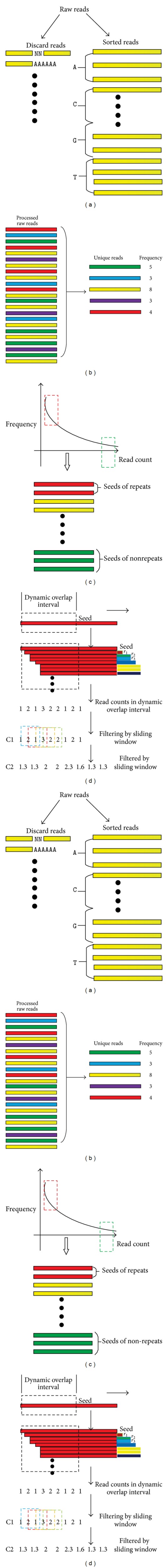
Some key steps of SWA. (a) Raw reads processing. Input reads containing any “*N*” or a low quality region are discarded and then sorted in alphabetical order. (b) Graphical illustration of unique process. The five different color lines represent the five unique reads in preprocessed raw reads. Each of them appears more than twice. By unique processing, the identical reads are collapsed into one unique and corresponding frequency. (c) Seed selection. The unique reads are ranked by read count (from high to low). Unique reads with read count larger than *H*_*p*_ are selected as seeds for repeat (the red dotted frame), while unique reads with read counts smaller than *L*_*p*_ are selected as seeds for nonrepeat extension (the blue dotted frame). (d) The graphical example of extending repeats using sliding window function in dynamic overlapping interval. The dotted box represents the dynamic overlapping interval *L*_*d*_. After overlapping with seed in *L*_*d*_, the overlapped read counts are recorded and then sliding window function is used to filter out the read bias in this interval continuously, as shown in C1. C2 is the corresponding results filtered by sliding window function, and then the mean value of this interval is recorded in variable *M*_*n*_ to detect the boundary of repeats (Figure 4) and nonrepeats (Figure 5). In this extension, SWA regards *r*_1_ as the optimal extendable read. The extension of nonrepeats is performed in a similar way. The detailed extension and boundary detection are graphically shown in Figures 4 and 5.

**Figure 3:**
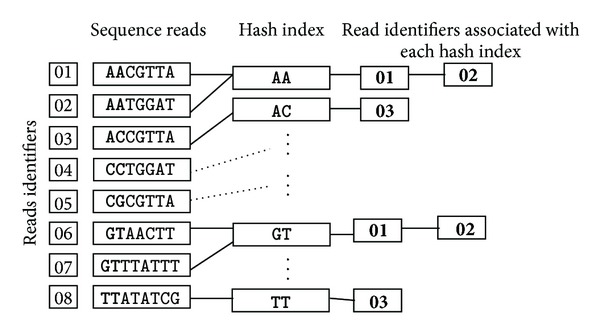
Schematic of a hash index of short sequences strategy. Sequence reads with associated read identifiers are shown, with the regions that will be used for seed selection in capital letters and matched seeds of two bases from AA to TT. Given read identifiers are associated with the seeds using a hash function (e.g., a unique integer representation of each seed). Once such hash table has been built for unique reads; the corresponding data can be scanned with the same hash function, resulting in a much smaller subset of reads to more exactly search the extendible reads.

**Figure 4:**
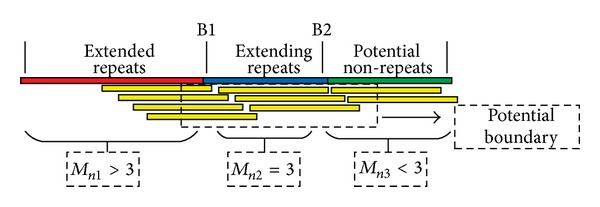
Schematic of extending repeats and boundary detection. The graphic illustration of extending repeats and boundary detection. Red line represents the extended repeats, blue line represents the extending repeats, and the green line represents the potential nonrepeats. The yellow lines represent the supporting reads overlapped with the extended contig. We assume that the sequencing depth *S*_*d*_ = 2, and let *T*_2_ = 3. Therefore, in the process of extending repeats, the mean value *M*_*n*_ of dynamic overlapping interval filtered by sliding window as shown in Figure 2(d) should be larger than or equal to *T*_2_. The dotted box represents the potential boundary of repeats. Consequently, if we set *M*_*n*_ > *T*_2_, the extension will be stopped at B1 or the extension will be stopped at B2.

**Figure 5:**
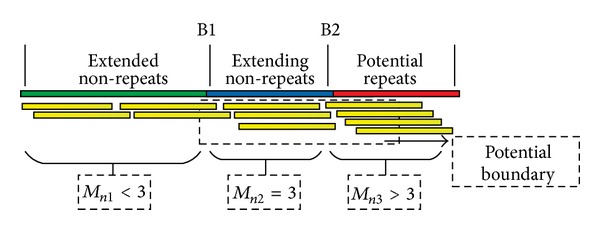
Schematic of extending nonrepeats and boundary detection. The graphic illustration of extending repeats and boundary detection. Green line represents the extended nonrepeats, blue line represents the extending nonrepeats, and the red line represents the potential repeats. The yellow lines represent the supporting reads overlapped with the extension. We assume that the sequencing depth *S*_*d*_ = 2, and let *T*_1_ = 3. Therefore, in the process of extending nonrepeats, the mean value *M*_*n*_ of dynamic overlapping interval filtered by sliding window should be smaller than or equal to *T*_1_. The dotted box represents the potential boundary of repeats. Consequently, if we set *M*_*n*_ < *T*_1_, the extension will be stopped at B1 or the extension will be stopped at B2.

SWA runs in five key steps (Figures 1 and 2): preprocessing, unique processing (Figure 2(b)), hash index (Figure 3), seed selection (Figure 2(c)), and seed extension (Figures 2(d), 4, and 5).

Firstly, preprocessing is performed. SWA firstly filters out the raw reads that contain any “*N*”, which is noninformative, or any low quality value region, which may contain errors and lead to false positive overlaps with other reads (Figure 2(a)). And then the parameters are set in advance (see parameters): the maximum overlapping length max⁡, dynamic overlapping interval *L*_*d*_, read length *L*_*r*_, length of sliding window *L*_*w*_, threshold of repetitive seeds *H*_*p*_, threshold of nonrepetitive seeds *L*_*p*_, threshold of repeats assembly *T*_2_, threshold of nonrepeats assembly *T*_1_, and sequencing depth after filtering by sliding window *S*_fd_.

Thirdly, unique processing is performed (Figure 2(b)), which is used to find the unique reads and corresponding frequencies in raw datasets. This step is performed using* unique* function provided by MATLAB platform. For example, *C* = unique ⁡(*A*) for the array *A* and returns the same values as in *A* but with no repetitions. The values of *C* are in sorted order. For the sequencing reads, this step is performed in a similar way. The reads that are exactly the same—that is identical reads—are collapsed into one unique read and the corresponding frequency is also recorded. For each raw read, the reverse-complement is also used. After unique processing, all unique reads are sorted in a table *R*, which is a variable to store these processed data. By unique processing, the amount of data is decreased, especially for the high coverage data, which can also reduce the computational requirement and accelerate the running time.

Thirdly, hash index is constructed (Figure 3). As it is time consuming to identify unique reads by directly comparing the whole raw reads one by one, SWA constructs the indirect hash index of unique reads which is able to index complex and nonsequential data in a way that facilitates rapid searching. The indirect hash index structures firstly transform the keywords to the quaternary integers and then index these integers rather than the strings directly. This is especially appropriate for DNA sequencing reads.

Fourthly, seed selection is conducted. In SWA, each extension requires a unique read, called a seed, to initiate the extension. In an extension-based assembler, a good seed should not contain any sequencing errors and should not be selected from the boundary of repeats and nonrepeats. Consequently, data cleaning is necessary in the data preprocessing stage before seed selection, and then the seeds are selected in table *R*. In addition, theoretically, a read from repetitive region usually has a high read count because identical repeats from other loci are counted as well; a read from nonrepetitive region always has a low read count. On the other hand, the read from the boundary always has the middle read count; these seeds always lead to misassembled and short contigs. Thus, the seeds for repeats are chosen with high read count in table *R*, larger than *H*_*p*_, while the one for nonrepeats should be chosen with low read count in table *R*, smaller than *L*_*p*_. And seeds with middle read count should be avoided. In order to avoid the risk of selecting seed with full errors, the lower limit of read count is also necessary. Furthermore, in seed selection stage, sequencing base quality value will be used to avoid the risk of picking seed with errors, especially for seed of nonrepeats. For the seeds with same read count, SWA selects the one with higher quality value, because higher base quality means lower errors. This strategy can avoid the risk of picking seed with errors to maximum extent.

Fifthly, seed extension (Figures 2(d), 4, and 5). We first define some terms. Let *L*_*r*_ be the length of each read and *x* and *y* be two unique reads in table *R*. We say that *x* and *y* overlap if the suffix *t* bases of *x* are identical to the prefix *t* bases of *y*, where min⁡≤*t* ≤ max⁡ (max⁡ and min⁡ are, resp., the allowed maximal and minimal numbers of overlapping bases) and the default value of them are *L*_*r*_/2 ≤ min⁡<max⁡=*L*_*r*_ − 1. More explicitly, we say that the 3′-end of *x* overlaps with the 5′-end of *y*. In the circumstances of long reads, the max overlap max⁡ is not needed to use this default value, and the empirical value is min⁡<max⁡<min⁡⁡{100, *L*_*r*_ − 1}. The dynamic overlapping interval *L*_*d*_, that is the *k*-mer in SWA, and is defined as *L*_*d*_ = *k*-mer = max⁡−min⁡. Given a seed, SWA first extends it at the 3′-end and then at the 5′-end. A read is extendable for *r*_seed_ if its 5′-end overlaps with the 3′-end of *r*_seed_ and its 3′-end overlaps with one or more unique reads. To extend a seed *r*_seed_ at the 3′-end, SWA searches all unassembled unique reads in table *R* for extendable reads in the dynamic range of [1, *k*-mer], that is, from maximal overlapping to minimal overlapping. The read counts overlapping with seed in this interval are recorded (as shown in Figure 2(d)). Then sliding window function is used to filter out the read count bias of this interval, and then the mean value *m*_*y*_ filtered by sliding window is recorded in variable *M*_*n*_ which is used to detect the boundary of repeats. For the extension of repeats (Figure 4), the extension will continue if *M*_*n*_ > *T*_2_, where *T*_2_ is the threshold of repeats, else the extension will be stopped at this end. For the extension of nonrepeats (Figure 5), the extension will continue if *M*_*n*_<*T*_1_, where *T*_1_ is the threshold of nonrepeats. Meanwhile, the mean value of variable *M*_*n*_ can be used to estimate the copy numbers.

In seed extending stage, dynamic overlapping interval *L*_*d*_ is used to search the optimal read to extend seed and which has three functions: (1) the optimal read can be searched in this interval for seed extension (Discussion); (2) the bias of the read counts in this interval can be filtered out by sliding window function so as to increase the confidence of detecting boundary (Figures 4 and 5); (3) copies of assembled contig can be estimated based on the combination of this interval. Moreover, sliding window is a determinant factor of judging whether the extension of seed is up to the bound of repeats or nonrepeats. The threshold for repeats and nonrepeats is closely related to the sliding window and filtered times, which determines the accuracy and correctness of repeat or nonrepeat contig construction.

The concrete contents and abbreviations are described in detail in methods and parameters.

### 2.2. Compensational Mechanism

Another property of SWA is the mechanism of compensating the loss caused by low sequencing depth. In practice, the genome sequencing process is highly uneven among the whole genome and full sequencing bias. High coverage can decrease the influence of sequencing bias on the statistical significance of read counts. However, under the condition of low coverage, sequencing bias reduces statistical significance of read counts, which leads to difficulty of distinguishing boundary of repeats. In order to improve the statistical significance of read counts under low coverage, sequencing bias will be filtered out more completely. To this end, SWA proposed a compensational mechanism by the combination of filtered times and sliding window function. The so-called filtered times *N*_*f*_ is the parameter of SWA which means the times of sequencing data filtered by sliding window function. For example, if the read counts filtered by sliding window function is filtered by sliding window once more, that is filtered times *N*_*f*_ = 2. The main idea of the compensational mechanism is as follows: SWA can increase the length of dynamic overlapping interval *L*_*d*_ and the size of sliding window *L*_*w*_ and then coalesce several points into one point, which can be achieved by both increasing the size of sliding window and number of filtered times.

The relationship between sequencing depth filtered by sliding window, filtered times *N*_*f*_, and average sequencing depth *S*_*d*_ is given as follows:

(1) $$\begin{array}{c} S_{\text{fd}}=S_{d}\times L_{w}^{N_{f}}. \end{array}$$

After filtering by sliding window, read counts will be more flat. To run this compensational mechanism, we suggest that only the values, size of sliding window, and filtered times, may need to be adjusted. The empirical value of optimal sliding window should be set in the range *k*-mer/5 ≤ *L*_*w*_ ≤ *k*-mer/3, where *k*-mer is the size of dynamic overlapping interval, and optimal filtered times *N*_*f*_ should be set *N*_*f*_ = 2.

Notably, this compensational mechanism is a double edge sword. On the one hand, it can decrease the sequencing bias and compensate the read loss of low coverage. On the other hand, it reduces the sensitiveness of detecting boundary of repeats and increases the computing complexity and executive time. In order to get the best assembly, the high coverage NGS data is also preferred.

### 2.3. Copy Number Estimation

The auxiliary function of SWA is to estimate the copy numbers of each assembled contig including repeats and nonrepeats directly from NGS data rather than aligning them back to reference genomes. Copy number estimation is also an important factor for the genomic function analysis related to CNVs. SWA estimates the copy number of the assembled contig when its extension is stopped at both ends. SWA output this finished contig and its corresponding copies simultaneously. Furthermore, the accuracy of estimated copy numbers is high up to 99% as shown in assessments in simulated datasets. Because in the process of extension of a seed, the mean value of each extension is recorded in variable *M*_*n*_, which is used to estimate the copy number of corresponding items (Methods).

## 3. Assessments

### 3.1. Metrics

In order to evaluate the ability of SWA for assembling repeats and nonrepeats independently, we use the metrics including Types of Repeat Contigs (TRC), Copy Number of each Repeat Contig (CNRC), Number of Nonrepeat Contigs (NNC), Number of Contig (Number C), N50, N90, Mean Contig, Maximum Contig, CN-accuracy, Rep-accuracy, C-accuracy, E-size, and Genome coverage.

TRC is the types of repeats and CNRC is the copy numbers of each corresponding type of repeat. These two metrics are used to evaluate the correctness of assembled repeats. NNC is the number of nonrepeat contigs which is used to evaluate the correctness of assembled nonrepeats. Because N50 size might sometimes be a misleading statistic, we also computed another statistic, which we called E-size.

- CN-accuracy is the accuracy of estimated copy number of each repeat. And which is designed to evaluate the accuracy of estimating copy number of each repeat contig and defined as CN-accuracy = 1 − (∑*N*_error_/*N*_total_), where *N*_total_ is the sum of the copy numbers of all types of repeats and *N*_error_ is the absolute value between estimated copy numbers and theoretical copy numbers. Therefore, the larger CN-accuracy is preferred. The larger the CN-accuracy is, the better the performances of SWA of estimating copy numbers of repeats will be.
- Rep-accuracy (Rep-acc) is the accuracy of assembled repeat contigs, which is designed to evaluate the correctness of all types of assembled repeat contigs and defined as Rep-accuracy = 1 − (∑|*L*_*a*_ − *L*_*r*_|/∑*L*_*r*_), where *L*_*r*_ represents the length of real repeat and *L*_*a*_ represents the length of assembled repeat contig. |*L*_*a*_ − *L*_*r*_| is the error tolerance. The higher Rep-accuracy represents the better performances of SWA for assembling repeat contigs. So higher Rep-accuracy is preferred.
- C-accuracy is the accuracy of the total contigs, which is designed to evaluate the accuracy of all assembled contigs totally and defined as C-accuracy = 1 − (*L*_error_/*L*_total_); *L*_total_ represents the total number of all contigs including repeats and nonrepeats and *L*_error_ is the sum of all error contigs.
- The E-size is designed to answer the question: if you choose a location (a base) in the reference genome at random, what is the expected size of the contig or scaffold containing that location? This statistic is one way to answer the related question: how many genes will be completely contained within assembled contigs or scaffolds, rather than split into multiple pieces? E-size is computed as E = ∑_*c*_(*l*_*c*_)^2^/*G*, where *l*_*c*_ is the length of contig *C* and *G* is the genome length estimated by the sum of all contig lengths.

For evaluating correctness, the metrics, such as CN-accuracy, Rep-accuracy, and C-accuracy, are all computed by aligning the corresponding items back to the reference genome using program* swalign* from MATLAB platform.* Swalign* constructs local pairwise alignments between two sequences using Smith-Waterman algorithm [25].

Among these metrics, TRC, CNRC, NNC, CN-accuracy, and Rep-accuracy are specially designed for judging the correctness of assembling repeats and nonrepeats. Notably, the length of contigs is not what we are mainly concerned about due to following reasons.

1. The primary goal of SWA is to resolve repeats by assembling repeats regions and nonrepeats regions separately. Therefore, the correctness of assembling repeats and nonrepeats is what SWA is firstly concerned about.
2. In order to separate repeat or nonrepeat correctly, SWA must detect the boundary of them accurately and stop extending seed at the boundary automatically.
3. The assembly with larger contig is always preferred. However, if the contig is assembled or constructed with errors, the larger the contig is, the worse the assembly will be. So accuracy is another important metric for the correct contig.

### 3.2. Assessments in Simulated Datasets

In this part, we validated the performances of SWA in three kinds of simulated datasets containing interspersed repeats, tandem repeats, and compound repeats, respectively. And then the effect of sequencing depth and read length to SWA was evaluated, respectively. The detailed results were shown in Tables 1, 2, and 3.

From Table 1, three kinds of simulated datasets containing interspersed repeats, tandem repeats, and compound repeats were used to validate the performances of SWA. The repetitive contents contained in these three sequences represented a wide range of repeats with different copies and lengths. The maximum of copy number and length of repetitive sequence are set up to 18 and 5 kb, respectively. The CN-accuracy, C-accuracy, and Rep-accuracy were almost up to 100% and 99.9%, respectively, which indicated that the estimated copy numbers of assembled repeats and the constructed contigs were all absolutely correct, and the error tolerance of constructed repeats was lower than 0.2%. The error rate of genome coverage was up to 1% low. All of these indicate that SWA not only can assemble different kinds of repeats and nonrepeats independently but also can estimate their copy numbers accurately.

From Table 2, we can clearly see that sequencing depth has a great influence on the performances of SWA. When depth = 6 or 4, the performances of SWA were so much better, metrics such as TRC, CNRC, CN-accuracy, and C-accuracy were absolutely correct and high. Rep-accuracy and N50 were a little down but still good; when depth dropped to 2 or 1, TRC and NNC were increasing, while N50, Max, and Rep-accuracy were decreasing; meanwhile the completeness of assembled repeats is getting worse. All of these indicated that the performances of SWA were degenerating; that is, long repeats and nonrepeats were assembled into several short fragments. CNRC of corresponding assembled repeat contig was shown in Appendix. But CN-accuracy and C-accuracy were still up to 100% which indicated that the copy number estimation of each assembled repeat contig was still right. When depth fell to 0.5, the metrics except CN-accuracy and C-accuracy were almost not accurate. Particularly, the Rep-accuracy was only 66.7%, which indicated that the completeness of repeats was destroyed. Notably, when depth dropped to 0.2, almost all metrics were getting bad. TRC and NNC were far from the real value. CN-accuracy and Rep-accuracy were so low that almost half repeats were not assembled and their copy numbers were estimated with large errors. N50 and Max were so small which indicated that all long repeats and nonrepeats were broken into smaller fragments. The assembled repeats and nonrepeats were far from completeness. But C-accuracy was more robust than other metrics, which indicated that although these contigs were so small they were at least correct. All of these indicate that sequencing depth affects the performances of SWA greatly. Some extent of high coverage depth is necessary for SWA to generate best assembly.

From Table 3, we can clearly see that read length has little influence on the performances of SWA. When read length varied from 36 to 150, almost all metrics were good and had little change except TRC and NNC. TRC and NNC were decreasing, which indicated that long repeats and nonrepeats were assembled more completely. Therefore, N50 increased a little with the increase of read length. What is more, the Rep-accuracy and genome coverage were increasing a little with the increasing of read length, which indicated the completeness of assembled repeats and assembly redundant were increasing simultaneously.

From Tables 1, 2, and 3, the property of assembling repeats and nonrepeats independently and separately was validated. The effect of sequencing depth and read length to the performances of SWA was also evaluated, respectively. From these results we can safely come to a conclusion that SWA can assemble repeats and nonrepeats independently and correctly; meanwhile sequencing depth has a greater influence on SWA than read length. In high coverage depth, the total performances of SWA are perfectly good. But in a low coverage depth situation, the performances of SWA are a little down. In practice, the higher coverage generated increases the higher sequencing cost. So a compensational mechanism of low sequencing depth is described in the following section.

In the following section, we evaluated the effect of sliding window and filtered times to SWA in low sequencing depth situation, respectively. The detailed results were shown in Tables 4 and 5.

Table 4 indicated that the performances of SWA were not so much good in low sequencing depth and sliding window can improve the performances of some metrics, such as TRC, NNC, and Rep-accuracy. When sliding window size *L*_*w*_ varied from 3 to 11, the performances of SWA were getting from good to bad, then to bad generally. Particularly for the metric of Rep-accuracy, which was getting up to 98.7% from 76.1% and then getting down to 89.6%. So the appropriate sliding window can improve the performances of SWA and compensate the read loss of low coverage depth. The choice of optimal sliding window is very important for the effect of compensational mechanism and is closely related to read length, sequencing depth, and dynamic overlapping interval.

It was clearly shown in Table 5 that the appropriate increase of filtered times could also improve the accuracy of assembling repeats. The effect of sliding window is to filter out the bias caused by sequencing process; therefore the increase of filtered times can improve the effect of filtering bias. It is clear that the Rep-accuracy was up to 82.1% after being filtered twice from 66.8% but got worse to 68% after three times filtering. So too much filtered times can lead to misassembled contigs across the boundary of repeats and nonrepeats as shown in Table 5. Therefore, for real NGS data, some compromise is necessary for choosing optimal filtered times.

### 3.3. Assessments in Reference Datasets

In this section, we validated the performances of SWA in reference genome datasets of three species in Materials. We analysed their repeat structures by whole genome scan using RepeatScout [26]. The repeats structures including lengths and copies were detailed in the Appendix and the link* detected repeats and their copies in Table* *6* in supporting data. The results of SWA are presented in Table 6.

Table 6 shows the assembly statistics of three species by SWA. All contigs were corrected and verified by aligning them back to reference genome, so the C-accuracy was 100%. For chrIV-S.c datasets, SWA generated 32 repeats and 315 nonrepeats; the copy numbers of assembled repeats are presented in the Appendix. In our whole genome scanning, 41 repeats longer than 200 were identified. By aligning these assembled repeats back to the reference, 4 tandem repeats were assembled together and the left were all correct. So the CN-accuracy is about 88%, but C-accuracy and genome coverage are almost up to 100%. For* E. coli*, SWA generated 50 repeats and 259 nonrepeats; the copy numbers of assembled repeats are presented in the Appendix. By whole genome scanning, 57 repeats were identified. So CN-accuracy is about 88%, but genome coverage is 99.7%. For chrIII-C.e datasets, SWA generates 198 repeats and 5471 nonrepeats. In our whole genome scanning, 339 repeats longer than 200 were identified. By aligning these assembled repeats back to reference, 103 short tandem repeats were assembled together. So the accuracy is about 89%. But the genome coverage is a little lower.

### 3.4. Assessments in NGS Datasets

In order to assess the performances of SWA more comprehensively, we performed comparisons with other eight leading genome assemblers presented in GAGE [27], such as ABySS, ALLPATHS-LG, Bambus2, CABOG, MSR-CA, SGA, SOAPdenovo, and Velvet. We used the assembly evaluation script provided by GAGE to assess various assembly metrics. Briefly, the GAGE script aligns contigs back to the reference genome and calculates the corrected N50 length by breaking contigs at misassembled sites. Tables 7, 8, and 9 show the assembly metrics for SWA and eight others in three species including* S. aureus*,* R. sphaeroides*, and human chromosome 14. We did not run these assemblers on the whole human genomes due to the following reasons: (1) some of the assemblers in our comparison would take many weeks to assemble the complete genome and others would fail entirely; and (2) high computing platform is not available. The statistics for these eight assemblers were taken from GAGE study. In order to compare the ability of assembling repeats fairly, all contigs are aligned back to repeats using* swalign* function [25].

Table 7 shows the assembly statistics for* S. aureus* dataset by nine assemblers. For* S. a*, SWA performs best in assembling repeats and nonrepeats. By aligning them back to repeats, SWA generated 30 repeats with total size 20.7 kb. SGA generated 18 repeats with total size 19.4 kb. Velvet generated 15 repeats with total size 13.8 kb. In terms of the completeness of types of repeats, SWA achieved the best assembly. The Rep-acc of SWA and SGA and Velvet are 82% and 83.8%, respectively. Therefore, for the accuracy of assembling repeats, SWA and SGA are the top two assemblers. Other assemblers had poor performances in the accuracy of assembling repeats and nonrepeats. Particularly, Allpath-LG only generates 3 repeats with size 2.6 kb and Rep-acc 4.3%. Because Allpath-LG achieved the longest corrected N50 length (66.2 kb), long contig can cross the boundary of repeat and lead to indistinguishable contig. So the better the continuity of assembler is the worse the completeness of assembling repeats and nonrepeats will be. For the continuity of assembly, SWA was read loss to other eight assemblers. However, for the assembly size and genome coverage, SWA was also the best one with assembly size 2,939 kb and genome coverage 100.1%, which is most approximate to the real genome size. So in terms of completeness of assembly, SWA achieved the best assembly.

Table 8 shows the assembly statistics for* R. sphaeroides* dataset by nine assemblers. By aligning them back to repeats, SWA generated 13 repeats with total size 7.8 kb. AByss, SGA, and SOAPdenovo generated 13 repeats with total size 8.3 kb, 9 repeats with total size 3.9 kb, and 9 repeats with total size 3.7 kb, respectively. The Rep-acc of them is 44.5%, 44.5%, 64.3%, and 84.5%, respectively. Therefore, in terms of accuracy of assembling repeats, SOAPdenovo outperformed others. But for the completeness of size of repeats, SWA and AByss are the top two assemblers. Other five assemblers performed worse in assembling repeats and nonrepeats. Particularly, Allpath-LG, Bambus2, and CABOG only generated less than three repeats with length less than 1.4 kb, because in terms of continuity, these three assemblers, Allpath-LG, Bambus2, and CABOG, are the top ones. But long contigs can lead to indistinguishable contigs. However, in terms of assembly size and genome coverage, SWA also achieved the best assembly with genome size 4,600 kb and genome coverage 99.9%, which is most approximate to the real genome size. So in terms of completeness of assembly, SWA performed best among these nine assemblers.

Table 9 shows the assembly statistics for human chromosome 14 dataset by nine assemblers. For* H. s* 14, SWA performs best in assembling repeats and nonrepeats. By aligning them back to repeats, there are four top assemblers in terms of assembling repeats, such as SWA, MSR-CA, SOAPdenovo, and Velvet. There are 198 repeats with total size 129.3 kb, 190 repeats with total size 526 kb, 188 repeats with total size 476 kb, and 192 repeats with total size 339 kb generated by SWA, MSR-CA, SOAPdenovo, and Velvet, respectively. The Rep-acc of corresponding items is 88.3%, 85.3%, 73.3%, and 56.9%, respectively. For the accuracy of assembling repeats, SWA and MSR-CA achieved the best results. In terms of completeness of types of repeats, SWA achieved the best results. However, in terms of continuity, Allpath-LG, CABOG, and SOAPdenovo outperformed SWA. But for the genome size and genome coverage, SWA achieved the best results with assembled size 8,7936 kb and genome coverage 99.6%. So in terms of completeness of assembly, SWA outperformed other assemblers.

One can safely come to a conclusion from Tables 7, 8, and 9 that SWA performed best in assembling repeats and nonrepeats in three NGS datasets. In terms of assembling repeats and completeness of repeats, SWA is the top one among these nine assemblers. One may argue that the contiguity of SWA is not better than others; the metrics such as N50, N90, and E-size are smaller than some of the other assemblers. This is because SWA is specially designed for assembling repeats and nonrepeats. Therefore, SWA stops extending contigs automatically when the boundary of repeats is detected. Meanwhile, the continuity of assembly and the completeness of repeats and nonrepeats are the pair of contradiction. On the other hand, the better completeness of repeats and nonrepeats requiers that contigs must be stopped at the boundary of repeats. Therefore, the continuity of assembly will be down.

## 4. Discussions

### 4.1. Sequencing Strategies for SWA

The uniformity of the sequencing process is very important for SWA, because assembling repeats and nonrepeats independently of SWA is based on the combination of coverage depth and sliding window. The effect of sliding window is to filter out the bias caused by sequencing process, because sequencing bias makes the frequency of repeat and nonrepeat more ambiguous to determine, so large sequencing bias may result in short contigs or misassembly. For the appropriately uniformed sequencing data, SWA cannot only assemble repeats and nonrepeats independently but can also estimate their copy numbers correctly. In this situation, contigs of repeats and nonrepeats can be easily grouped into scaffolds by SWA using only the short insert paired-end information, while other current assemblers all need the good combination of several mate-pair libraries with different insert lengths, which increase the cost of sequencing and complexity of technologies. Therefore SWA provides a simple sequencing strategy for NGS technologies; that is, long-distant library is not necessary. So similar to the strategies recommended by ALLPATHS-LG [19], we recommend that for the Illumina technology one should use an overlapping paired-end library with a suitable insert size to generate PE raw reads for contig assembly and there is no need for several mate-pair libraries with different insert lengths to generate long-distant jumping reads for scaffolds. The average genome coverage is at least 100× or higher. For generating overlapping paired-end reads, we provide a simple formula to calculate the insert size for constructing a paired-end library: insert Size = (read length (*l*)  + max error tolerance (*m*)) × 2 − max overlap length (*n*). For example, if the read length is *l* = 150 bp, the max overlap length *n* = 100 bp and the max error tolerance *m* = 50; then the recommended insert size is 300 bp.

### 4.2. Seed Selection

In an extension-based assembler, a good seed should not contain any sequencing errors and should not be selected from the boundary of repeats and nonrepeats. A read from repeat region usually has a high read count because identical repeats from other loci are counted as well. On the other hand, a read from nonrepeat region always has a low read count. However, the read from the boundary always has the middle count under the condition of uniformed sequencing process. These seeds are hard to determine whether they belong to repeat region or nonrepeat region and always lead to misassembly or short contigs. Thus, the seeds for repeat region are chosen with high read count, while the one for nonrepeat region should be chosen with low read count, and seeds with middle read count should be avoided.

### 4.3. Parallel Operation

Obviously, parallel operation can save the executive time and reduce the memory use. SWA can assemble repeats and nonrepeats independently by using two different computers without any communication. This property can shorten the executive time almost a half and reduce the memory use in some extent. Furthermore, the raw data also can be classified into two parts, repeats and nonrepets, according to read count. This strategy can reduce the memory usage largely.

### 4.4. Optimal Sliding Window

The sliding window plays an important role in contig construction in SWA. By filtering out sequencing bias, SWA can distinguish repeats and nonrepeats from NGS data easily so as to assemble repeats and nonrepeats independently. The mean value of read count in sliding window determines whether the extension should continue or not. So too small sliding window cannot filter out the bias efficiently but has high sensitiveness of detecting changes of read count. A too large sliding window can filter out the bias efficiently but decreases the sensitiveness of detecting repeats and leads to misassembly. The optimal sliding window should have both the property of filtering bias efficiently and detecting repeats sensitively. So the compromise is necessary in practice.

### 4.5. Optimal Read

In the stage of seed extension, the optimal read is needed in order to extend the seed in dynamic overlapping interval.

In SWA, the optimal read was identified using the following strategy: the one overlapped most bases with seed in dynamic overlapping interval was taken as the optimal read. In theory, longer overlapping with seed means higher accuracy of assembly. But this strategy has low speed, because the seed only extends one or two bases at one extension. Of course, the other strategy for choosing optimal read in dynamic overlapping interval also can be adopted, such as the optimal read can be identified as the one with read counts nearest to the theoretically sequencing depth. This strategy has a higher speed, but the correctness of extension will be low compared with the first strategy. In practice, the strategy of identifying optimal read can be chosen by users.

### 4.6. Comparisons

SWA is specially designed for assembling repeats and nonrepeats, respectively. What we are mainly concerned with is the correctness and accuracy of assembling repeats and estimating their copy numbers rather than the length of assembled contigs, so SWA stops extending contig automatically when detecting the boundary of repeat and nonrepeat. Duo to this, the validations and evaluations are performed rather than comparisons with other assemblers in simulations and reference datasets. In real NGS datasets, the comparisons were performed comprehensively with other eight leading assemblers. But the accuracy and completeness of repeats are what we are firstly concerned with.

## 5. Conclusions

In this paper, we developed a* de novo* genome assembly algorithm named SWA, which can assemble repeats and nonrepeats independently. The most important features of SWA are (1) assembling repeats and nonrepeats completely and accurately; (2) adopting sliding window function to filter out sequencing bias in genome assembly process; (3) compensating the loss of low coverage; and (4) estimating the copies of each assembled contigs. Consequently, in this study, we have validated the performances of SWA and compared them with other leading assemblers in three real NGS datasets. For comparisons in real NGS datasets, the metrics such as TRC, NNC, and Rep-size are used to evaluate the completeness of assembled repeats and nonrepeats; the metrics such as Rep-accuracy, C-accuracy, and CN-accuracy are used to evaluate the accuracy of assembled repeats and nonrepeats, while the N50, N90, and maximum contig are used to evaluate the continuity of whole genome assembly. Results indicated that SWA outperformed other leading assemblers in the completeness and correctness of assembling repeats, but the continuity was not better than some of the others. It is natural, because SWA is not specially designed for whole genome assembly and continuity is not what SWA is firstly concerned with.

In general, without long insert-size libraries, repeats that extend beyond the paired-end insert sizes will be difficult to resolve and assemble. Although, some of the compared assemblers can assemble long repeats with simple structure, the completeness and accuracy are not good. It is natural that the continuity is what a whole genome assembler is mainly concerned with. However, SWA is not a whole genome assembly. So bridging two unique sequences around a repeat is not allowed by SWA in order to ensure the completeness of separating repeats and nonrepeats. Even though SWA was not aiming for the whole genome assembly, SWA also provided another solution to resolve long repeats without the help of long insert-size libraries by assembling from nonrepeats completely. In theory, for the whole genome assemblers, if repeats and nonrepeats are assembled correctly and completely, their copies are estimated correctly. Scaffolds can be grouped easily by using short-insert paired-end information rather than the good combination of several libraries. In practice, repeat characteristics in different genomes can vary extensively and depths of sequencing can be highly uneven along the genome, so the expected theoretical* de novo* assembly results from different genomes will also vary.

## 6. Methods

### 6.1. The Detailed Outlines

#### 6.1.1. The Steps of Extending Repeats (Figure 4)

1. Selecting a seed in repeat regions with high frequency larger than *H*_*p*_ in table *R*.
2. Computing read counts overlapped with seeds in dynamic overlapping intervals.
3. Filtering the overlapped read counts by sliding window and then computing the mean value of this interval and recording in *M*_*n*_.
4. Judging whether the extension of seed is out of the bound of repeat or not. If *M*_*n*_ > *T*_2_, the extension will continue or else stop extension at this end.
5. Extending seed using the optimal read in the dynamic overlapping interval. The optimal read in SWA is the unique read with longest overlapped bases.
6. Continue Step 2–Step 5 until this seed is stopped at both 3′-end and 5′-end.
7. If repetitive seed sets are not empty, go to Step 1 and repeat these steps, else repetitive contigs construction are finished.

#### 6.1.2. The Steps of Extending Nonrepeats (Figure 5)

1. Selecting a seed in nonrepeat regions with low frequency smaller than *L*_*p*_ in table *R*.
2. Computing read counts overlapped with seeds in dynamic overlapping intervals.
3. Filtering the overlapped read counts by sliding window and then computing the mean value of this interval and recording in *M*_*n*_.
4. Judging whether the extension of seed is up to the boundary of repeat or not. If *M*_*n*_ < *T*_1_, the extension will continue, or else stop extension at this end.
5. Extending seed using the optimal read in the dynamic overlapping interval. The optimal read in SWA is the unique read with longest overlapped bases.
6. Continue Step 2–Step 5 until the extension is stopped at both 3′-end and 5′-end.
7. If nonrepetitive seed sets are not empty, go to Step 1 and repeat these steps, else nonrepetitive contigs construction are finished.

### 6.2. Sliding Window

Coverage bias is inevitable in genome sequencing process and is usually caused by whole genome amplification (WGA) [28]. Three primary forms of WGA have been developed: multiple displacement amplification (MDA) [29], primer extension preamplification (PEP) [30], and degenerate oligonucleotide primed PCR (DOP) [31]. Furthermore, coverage bias also can be amplified by data cleaning and error correction stage. The existence of coverage bias increases the nonuniformity of read depth for detecting copy numbers of repeats, which can lead to extending contigs crossing the bound of repeats and incorrect estimation of copy numbers of repeat contigs. How to eliminate or decrease these noises is a very important step in constructing contigs of repeat or nonrepeat regions. So the sliding window is used to filter out the noise caused by coverage bias so as to improve the performances of distinguishing repeats from nonrepeats and estimating the copy number of each repeat contig.

In order to decrease the coverage bias, we use rectangular window function to smooth coverage bias. Rectangular window function is defined as follows:

(2) $$\begin{array}{c} w\left(n\right)=\{\begin{array}{cc} 1, & 0\leq n\leq L_{w}-1\\ 0, & \text{otherwise}, \end{array} \end{array}$$

here *L*_*w*_ is the length of sliding window. So

(3) $$\begin{array}{c} w\left(i\right)=\frac{1}{L_{w}}\overset{i+(L_{w}/2)}{\underset{j=i-\left(L_{w}/2\right)}{\sum }}x_{j},\;i=1,2,\ldots ,L_{d}, \end{array}$$

where *x*_*j*_ represents read counts by overlapping *L*_*r*_ − *j* bases.

In theory, the longer the *L*_*w*_ is, the better the sliding effect will be. However, *L*_*w*_ cannot be too large or too small which is closely bounded to read length *L*_*r*_ and length of dynamic overlapping interval *L*_*d*_. In order to guarantee the correctness of SWA, we set *L*_*d*_ ≥ (3/4)*L*_*r*_, 2 ≤ *L*_*w*_ ≤ *L*_*d*_/2. Of course, this is an empirical value.

### 6.3. The Effect of Window Function

By mathematical derivation, we can clearly see that the variances smoothed by window function are smaller than the original ones. Letting *X*_*n*_ = {*x*_1_, *x*_2_,…, *x*_*n*_} be the *n* preprocessed data points and *E*(*X*_*n*_) = (1/*n*)∑_*i*=1_^*n*^ *x*_*i*_ be the mean of *X*_*n*_, the variance of *X*_*n*_ is as follows: *D*(*X*_*n*_) = (1/*n*)∑_*i*=1_^*n*^(*x*_*i*_−*E*(*X*_*n*_))^2^. Letting sliding window function *y* = *w*(*x*), *Y*_*n*_ = {*y*_1_, *y*_2_,…, *y*_*n*_} be corresponding smoothed data points, *y*_*i*_ = *w*(*x*_*i*_) = (1/*L*_*w*_)∑_*j*=*i*−*Lw*/2_^*i*+*Lw*/2^ *x*_*i*_, the mean value of *Y*_*n*_ is *E*(*Y*_*n*_) = (1/*n*)∑_*i*=1_^*n*^ *y*_*i*_. It is clear that *E*(*X*_*n*_) = *E*(*Y*_*n*_); *D*(*Y*_*n*_) = (1/*n*)∑_*i*=1_^*n*^(*y*_*i*_−*E*(*Y*_*n*_))^2^. One can easily prove that *D*(*Y*_*n*_) ≤ (1/*L*_*w*_)*D*(*X*_*n*_).

### 6.4. Hash Index

Overlap computing is the most time consuming stage for all against all. In order to speed up SWA, we use hash index to store the location of each keyword and read rather than the keyword itself in hash index. The details are as follows. For each read, the *N* continuous bases from 3′-end and 5′-end are selected and then mapped into two variables, forward and backward, respectively. And then, map *A* → 0, *C* → 1, *G* → 2, *T* → 3. So a string consisting of *N* continuous bases is transferred into quaternary integers; the quaternary integers then are transferred into decimal integer. So each keyword is mapped to a unique location in hash index which stores the identification of unique processed reads. We define the hash function as

(4) $$\begin{array}{c} H\left(S\left[i,i+L_{k}\right]\right)=Q_{i}Q_{i+1},\ldots ,Q_{i+L_{k}}\left(\text{quaternary}\right), \end{array}$$

where

(5) $$\begin{array}{c} Q_{i}=\{\begin{array}{cc} 0, & s\left[i\right]=\text{“}A\text{”}\\ 1, & s\left[i\right]=\text{“}C\text{”}\\ 2, & s\left[i\right]=\text{“}G\text{”}\\ 3, & s\left[i\right]=\text{“}T\text{”}; \end{array} \end{array}$$

*L*_*k*_ is the length of keywords in hash function.

### 6.5. Parameters of Kernel SWA Program

The kernel program of SWA has eight parameters: the maximum overlapping length max⁡, dynamic overlapping interval *L*_*d*_, read length *L*_*r*_, length of sliding window *L*_*w*_, threshold of repetitive seeds *H*_*p*_, threshold of nonrepetitive seeds *L*_*p*_, threshold of repeats assembly *T*_2_, threshold of nonrepeats assembly *T*_1_, and sequencing depth after filtering by sliding window *S*_fd_. On the basis of the extensive comparisons of three species as shown in the paper, we suggest that the only values of maximum overlapping length, length of dynamic overlapping interval, and read length may not be adjusted. We recommend the following: *L*_*r*_ = 101, max⁡ = 100, *L*_*w*_ = 9, and *k*-mer can be set in the range [35, 50]. These four parameters: *H*_*p*_, *L*_*p*_, *T*_1_, and *T*_2_ are closely related to sequencing depth *S*_*d*_ and length of sliding window *L*_*w*_. For example, if sequencing depth is *S*_*d*_ = 2, length of sliding window *L*_*w*_ = 3 and filtered times *N*_*f*_ = 1, and then *H*_*p*_ should be higher than double sequencing depth; that is, *H*_*p*_ = 5. *L*_*p*_ should be lower than sequencing depth, so let *L*_*p*_ = 1. *T*_1_ should be a little higher than the filtered sequencing depth *S*_fd_ = *S*_*d*_ × *L*_*w*_^*N*_*f*_^ = 2 × 3 = 6, so let *T*_1_ = 8. *T*_2_ should be a little lower than the 2 × *S*_fd_ = 2 × 6 = 12, so let *T*_2_ = 10, where sequencing depth is *S*_*d*_ = *N*_*r*_/*L*, coverage is *C* = (*N*_*r*_/*L*) × *L*_*r*_ = *S*_*d*_ × *L*_*r*_, and *N*_*r*_ is the number of reads.

Parameters have a great influence on the performances of SWA. Therefore, in practice, fine tuning is necessary for special genome with intrinsic complex repeat structure or different backgrounds of sequencing bias. We suggest that the parameters needed to fine tune are *T*_1_ and *T*_2_. The abbreviations are presented in the Abbreviation Section.

### 6.6. Thresholds

The threshold for repeats and nonrepeats are based on the size of confidence intervals and significance testing, which are closely related to coverage depth, size of sliding window, and filtered times.

Let *X* = {*x*_1_, *x*_2_,…, *x*_*n*_} be the overlap numbers in dynamic overlapping stage and the mean of *X* is *m*_*x*_ = (1/*n*)∑*x*_*i*_. Let *Y* = {*y*_1_, *y*_2_,…, *y*_*n*_} be the read counts filtered by sliding window, so the mean of *Y* is *m*_*y*_ = (1/*n*)∑*y*_*i*_ ≈ *m*_*x*_ × *L*_*w*_. According to the law of large number in the probability and statistic theory, we can easily get *Y* ~ *N*(*m*_*y*_, *σ* ^2^). So if the average sequencing depth is *S*_*d*_, the average read counts filtered by sliding window is *S*_fd_ = *S*_*d*_ × *L*_*w*_. Thus, the read counts of the nonrepeat region filtered by sliding window should be *S*_fd_ if sequencing bias is free, and the corresponding items of repeat region with two copies should be 2*S*_fd_. Clearly *S*_fd_ = *m*_*y*_ if sequencing bias is free. Let *δ* = *F*_*d*_/2; random variable *m*_*y*_ is the mean read counts filtered by sliding window.

If *P*{*m*_*y*_ ≤ *F*_*d*_ + *δ*_1_} ≥ 1 − *α*, so the confidence upper limit *T*_1_ of nonrepeat region at the confidence level 1 − *α* is *T*_1_ = *F*_*d*_ + *δ*_1_ which is the threshold of nonrepeats. If *P*{*m*_*y*_ ≥ 2*F*_*d*_ − *δ*_2_} ≥ 1 − *α*, the confidence lower limit *T*_2_ of nonrepeat region at the confidence level 1 − *α* is *T*_2_ = 2*F*_*d*_ − *δ*_2_ which is threshold of repeats, where 0 ≤ {*δ*_1_, *δ*_2_} ≤ *δ*, and {*δ*_1_, *δ*_2_} can be used to control the type-I error and type-II error since the statistical tests of overlapping intervals of windows are not independent. The construction of repeat contig and nonrepeat contig is generated separately.

### 6.7. Estimating Copy Numbers

The copy number of each repeat contig is estimated by using significant testing methods. After finishing contig construction, the variable *M*_*n*_ has stored the whole filtered read counts and its mean value is computed. The copy number of corresponding items is estimated by rounding mean(*M*_*n*_)/*S*_fd_ to the nearest integer.

## 7. Materials

In this study, three kinds of datasets are used to validate the performances of SWA and compare with other assemblers. They are real simulated datasets, reference datasets, and real NGS datasets.

**Figure 6:**
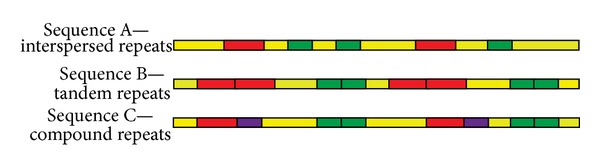
The graphic explaining the real simulated datasets. The yellow lines represent the model of random sequences. The red lines, blue lines, and purple lines represent different contents of repeats.* Sequence A *represents the interspersed repeats that is different repeats do not link each other closely.* Sequence B* represents tandem repeats that is same repeats link each other in the cascade manner.* Sequence C* contains the compound repeats, which is the combination of* sequence A *and* sequence B*. The detailed generation process is presented in supplementary materials.

For real simulated datasets, the model sequences, A, B, and C are randomly sampled from {A, T, C, and G} with different repetitive contents. These three sequences contain tandem repeats, interspersed repeats, and compound repeats, respectively (as shown in Figure 6). These repetitive contents represent a wide range of length and copies. The detailed information is presented in supplementary table (see Table S3 in Supplementary Material available online at http://dx.doi.org/10.1155/2014/736473). And the generation process is also presented in supplementary materials. Then, the paired-end NGS reads are randomly sampled from the fragments with normal distribution *N* (300, 30).

For reference genome datasets, we download the reference genome of* S. cerevisiae*,* C. elegans* from UCSC (http://hgdownload.soe.ucsc.edu/downloads.html) and* E. coli k12* (GenBank: U00096.3). For* S. cerevisiae* and* C. elegans*, we only randomly take chromosome IV and chromosome III, respectively. The sizes of chrIV-S.c,* E. coli*, and chrIII-C.e are 1,531,933 bp, 5,132,068 bp, and 13,783,700 bp, respectively. Their repeats structures can be easily analyzed by RepeatScout [26], which is a very effective and sensitive* de novo* repeats identification method for large genomes and is freely available at http://bix.ucsd.edu/repeatscout/. The repeats structures including lengths and copies are detailed in the Supplementary Materials Appendix and are freely available at http://222.200.182.71/swa/Table6.rar.

For real NGS datasets, two bacterial genomes (*Staphylococcus aureus* and* Rhodobacter sphaeroides*, genome sizes of 2.9 and 4.6 Mb, resp.) and* human chromosome 14* (genome size of 88.3 Mb) were downloaded from http://gage.cbcb.umd.edu/data/. In the GAGE study [27], all reads were error-corrected before assembly by ABySS, ALLPATHS-LG, Bambus2, Celera Assembler with the Best Overlap Graph (CABOG), Maryland Super-Reads Celera Assembler (MSR-CA), SGA, SOAPdenovo, and Velvet. For a fair comparison, we also obtained these corrected datasets for using in GAGE. These three species have perfect reference genomes. Therefore, their real repeats structures can be easily detected by RepeatScout [26]. For* S. a*,* R. s*, and* H. s* 14, there are 52 repeats, 21 repeats, and 259 repeats detected by RepeatScout [26], respectively, with length longer than 100 bp; their total sizes are 15.8 kb, 3.6 kb, and 146.5 kb, respectively.

### 7.1. Implementation

Table 10 presents the detailed memory usage and CPU times of SWA in three real NGS datasets. Different stages have different requirements of memory. The memory usage presented in Table 10 is the maximum memory. The CPU times refer to the run time of main procedure except for the preprocessing stage. Because a different assembler has different hardware requirements; therefore the direct comparisons are not reasonable to some extent. However, Table 10 gives users a rough guidance for hardware requirement and run time. Those of others have been accessed clearly in GAGE study and are freely available at http://genome.cshlp.org/content/early/2012/01/12/gr.131383.111/suppl/DC1.

SWA is implemented in MATLAB computing environment. Programming language: m language. Operation systems: Windows, Linux. Computing platform: 3.5 GHz eight Intel Celeron CPU with 32 GB RAM and 64-bit operational system.

## 8. Availability of Supporting Data

The supporting data including NGS data and assembling results are freely available at http://222.200.182.71/swa/Results.rar.

The detected repeats and their copies of three species used in Table 6 can be freely found at http://222.200.182.71/swa/Table6.rar.

The detected repeats and their copies of three species used in Table 7, 8, 9 can be freely found at http://222.200.182.71/swa/Tables789.rar.

## Supplementary Material

The supplementary materials include three parts: the first part is the performances of SWA in two species with extremly different CG contents presented in S-Table 1 and S-Table 2. These two species contained completely differnet CG contents. The results indicated that CG contents has little influences on the performances of SWA; the seconde part is the detailed generation process of three real simulated sequences in MATLAB platform; and the final part is the Appendix which contains the copy number information presented in Table 1-Table 6 and S-Table 1.

## Figures and Tables

**Table 1:** The performances of assembling different kinds of repeats.

Sequence (Containing)	Repeat			Contigs (kb)		Accuracy (%)			Genome coverage (%)
	TRC	CNRC	NNC	N50	Max	CN-accuracy	Rep-accuracy	C-accuracy	
Interspersed repeats	6	3, 6, 8, 5, 10, 8	73	9.1	26	100	99.9	100	100.9
Tandem repeats	6	6, 18, 9, 4, 20, 9	51	13.2	31.3	100	99.8	100	101
Compound repeats	12	Appendix	110	13.9	32.9	100	99.8	100	101

Three sequences with length *L* = 500 kb, 500 kb, and 1 Mb containing different types of repeats. Contigs of repeat and nonrepeat are generated independently by SWA with basic parameters: read length *L*_*r*_ = 50, filtered times = 1, sliding window size *L*_*w*_ = 3, and *k*-mer = 10. Contigs smaller than 200 are removed.

**Table 2:** The effect of depth to SWA.

Sequencing depth	Repeat			Contigs (kb)		Accuracy (%)			Genome coverage (%)
	TRC	CNRC	NNC	N50	Max	CN-accuracy	Rep-accuracy	C-accuracy	
6	5	3, 4, 2, 6, 5	21	19	48	100	99.9	100	100.4
4	5	3, 4, 6, 5, 2	25	19	48	100	99.9	100	100.4
2	9	Appendix	100	7.2	20.8	100	89.2	100	101.1
1	15	Appendix	323	2.1	11.8	100	86.7	100	101.9
0.5	14	Appendix	950	0.3	3.0	100	66.7	100	63.3
0.2	17	Appendix	54	0.2	1.0	80	56.7	100	8.3

Sequence length *L* = 500 kb containing five types of repeats. Sequencing depths are changing from 6 to 0.2. Contigs of repeat and nonrepeat are generated in an independent ways by SWA at different depths with basic parameters: read length *L*_*r*_ = 60, filtered times = 1, sliding window size *L*_*w*_ = 3, and *k*-mer = 10. Contigs smaller than 200 are removed.

**Table 3:** The effect of read length to SWA.

Read length	Repeat			Contigs (kb)		Accuracy (%)			Genome coverage (%)
	TRC	CNRC	NNC	N50	Max	CN-accuracy	Rep-accuracy	C-accuracy	
36	10	Appendix	33	19	34	100	98.2	100	100.1
50	9	Appendix	23	19	48	100	99	100	100.3
101	7	3, 4, 6, 5, 2, 2, 2	21	19.1	48.1	100	99.2	100	100.8
150	6	3, 4, 2, 6, 5, 2	22	19.3	48.3	100	99.6	100	101

Sequence length *L* = 500 kb containing five types of repeats. Read length is changing from 36 to 150. Contigs of repeat and nonrepeat are generated in an independent ways by SWA at different levels with basic parameters: sequencing depth *S*_*d*_ = 4, filtered times = 1, sliding window size *L*_*w*_ = 3, and *k*-mer = 10. Contigs smaller than 200 are removed.

**Table 4:** The effect of sliding window to SWA.

Window size	Repeat			Contigs (kb)		Accuracy (%)			Genome coverage (%)
	TRC	CNRC	NNC	N50	Max	CN-accuracy	Rep-accuracy	C-accuracy	
3	11	Appendix	1016	0.4	3.0	100	76.1	100	81.2
7	10	Appendix	688	1	4.0	100	98.7	100	101
11	15	Appendix	612	1.1	4.0	100	89.6	100	101

Sequence length *L* = 500 kb containing four kinds of repeats. Size of sliding window varies from 3 to 11. Contigs of repeat and nonrepeat are generated in an independent way by SWA at different levels with basic parameters: sequencing depth *S*_*d*_ = 0.5, filtered times = 1, read length *L*_*r*_ = 60, and *k*-mer = 20. Contigs smaller than 200 are removed.

**Table 5:** The effect of filtered times to SWA.

Filtered times	Repeat			Contigs (kb)		Accuracy (%)			Genome coverage (%)
	TRC	CNRC	NNC	N50	Max	CN-accuracy	Rep-accuracy	C-accuracy	
1	11	Appendix	801	0.3	2.3	100	66.8	100	53.4
2	12	Appendix	418	1.8	7.6	100	82.1	100	101.9
3	13	Appendix	1025	0.3	2.3	100	68	100	77.4

Sequence length *L* = 500 kb containing five kinds of repeats. Contigs of repeat and nonrepeat are generated in an independent way by SWA at different levels with basic parameters: sequencing depth *S*_*d*_ = 0.5, size of sliding window *L*_*w*_ = 3, read length *L*_*r*_ = 60, and *k*-mer = 15. Contigs smaller than 200 are removed.

**Table 6:** The results of SWA in reference genome datasets.

Species	Repeat			Contigs (kb)		Accuracy (%)			Genome coverage (%)
	TRC	CNRC	NNC	N50	Max	CN-accuracy	Rep-accuracy	C-accuracy	
chrIV-S.c	32	Appendix	315	9.4	32.7	88	93	100	100
E. coli	50	Appendix	259	46.5	190.5	88	99.8	100	99.7
chrIII-C.e	198	Appendix	5471	4.6	26.7	89	92	100	97.7

Contigs of repeat and nonrepeat are generated in an independent way by SWA with basic parameters: sequencing depth *S*_*d*_ = 2, read length *L*_*r*_ = 60, filtered times = 1, and sliding window = 3. Contigs smaller than 200 are removed.

**Table 7:** Assemblies of *S. aureus* (genome size 2,903,081).

Assemblers	Repeat				Contigs (kb)					*E*-size	Assembly size (kb)	Genome coverage (%)
	TRC	NNC	Rep-size	Rep-acc	Num.C	N90	Mean	N50	Max			
SWA	30	1834	20.7 kb	82%	1864	0.8	1.6	2.5	14.4	3115	2939	100.1
ABySS	7	293	6.8 kb	30%	302	7.0	12	24.8	125	31403	3647	125.6
Allpaths-LG	3	57	2.6 kb	4.3%	60	31	47	66.2	234	90078	2869	98.8
Bambus2	6	103	7.5 kb	35%	109	11	15	16.7	158	19610	2833	97.6
MSR-CA	7	87	4.4 kb	15%	94	21	30	48.2	139	50381	2862	98.5
SGA	18	1232	19.4 kb	83.8%	1252	1.0	2.2	4.0	16.8	4712	2833	97.6
SOAPdenovo	9	98	7.5 kb	38.6%	107	35.5	27	62.7	518.7	68002	2909	100.2
Velvet	15	147	13.8 kb	39.3%	165	11.4	17.6	41.5	169	48511	2847	98

N50, N90, and mean values are based on the same genome size. The contigs are all corrected and those smaller than 200 were removed.

**Table 8:** Assemblies of *Rhodobacter sphaeroides* (genome size 4,603,060).

Assemblers	Repeat				Contigs (kb)					*E*-size	Assembly size (kb)	Genome coverage (%)
	TRC	NNC	Rep-size	Rep-acc	Num.C	N90	Mean	N50	Max			
SWA	13	1774	7.8 kb	44.5%	1787	1.3	2.6	4.2	29.4	5812	4600.3	99.94
ABySS	13	1910	8.3 kb	44.5%	1915	1.1	2.5	4.2	54.7	6877	4969.5	108
Allpaths-LG	2	202	1.4 kb	6.7%	204	11.5	22.5	34.4	106	35973	4587.8	99.6
Bambus2	2	175	1.3 kb	9.2%	177	6.1	8.5	12.8	279	16281	4371.6	94.9
CABOG	1	312	0.3 kb	8.9%	322	6.5	13	17.9	88.5	21539	4238	92
MSR-CA	7	388	4.1 kb	25.4%	395	5.7	11.3	19	83.7	21579	4465	97
SGA	9	3053	3.9 kb	64.3%	3067	0.66	1.4	2.9	29.5	4067	4502.7	97
SOAPdenovo	9	195	3.7 kb	84.5%	204	7.8	11.8	14.3	376	18553	4596	99.8
Velvet	5	578	2.2 kb	24.5%	583	4.6	7.7	14.5	60.7	16711	4503	97.8

N50, N90, and mean values are based on the same genome size. The contigs are all corrected and those smaller than 200 were removed.

**Table 9:** Assemblies of human chromosome 14 (genome size 88,289,540).

Assemblers	Repeat				Contigs (kb)					*E*-size	Assembly size (kb)	Genome coverage (%)
	TRC	NNC	Rep-size	Rep-acc	Num.C	N90	Mean	N50	Max			
SWA	198	26824	129.3 kb	88.3%	27021	1.6	3.3	6.7	52	8737	87936	99.6
ABySS	143	51647	121.4 kb	30.1%	51924	0.7	1.7	2.0	30	3134	73341	83
Allpaths-LG	88	4441	156.7 kb	47.3%	4529	9.7	18.7	21.0	240	27157	84435	95.6
Bambus2	154	13437	293 kb	66.7%	13592	2.4	3.1	4.3	261	6345	68243	77.3
CABOG	70	3291	255 kb	39.9%	3361	13.7	21.6	23.7	296	30689	86232	97.6
MSR-CA	190	29901	526 kb	85.3%	30103	1.2	2.7	4.3	53.9	5927	83291	94.3
SGA	187	56278	283 kb	50.4%	56939	0.6	1.4	2.7	30	3737	82375	93.3
SOAPdenovo	188	21552	476 kb	73.3%	22689	1.8	4.2	7.4	141	9801	92603	104.9
Velvet	192	45294	339 kb	56.9%	45564	0.7	1.6	2.1	22.5	3049	74740	84.6

N50, N90, and mean values are based on the same genome size. The contigs are all corrected and those smaller than 200 were removed.

**Table 10:** Memory usage and CPU times of SWA.

Species	Memory usage	CPU times
*S. aureus *	2.5 GB	59.5 minutes
*Rhodobacter sphaeroides *	3.4 GB	96.3 minutes
Human chromosome 14	22.6 GB	56.2 hours

## Abbreviations

SWA: Sliding window assembling
TRC: The types of repetitive contigs
CNRC: Copy number of each repeat contig
NNC: Number of nonrepeat contigs
Rep-acc: The accuracy of assembled repeat contigs
C-accuracy: The accuracy of the total contigs
CN-accuracy: The accuracy of estimated copy number of each repeat
*k*-mer: Maximum overlap minus the minimum overlap
*N*_*f*_: The number of filtered times by sliding window
*L*_*w*_: The length of sliding window
*L*_*r*_: The length of reads
*L*_*d*_: The length of dynamic overlapping interval
*H*_*p*_: The threshold of repetitive seeds
*L*_*p*_: The threshold of nonrepetitive seeds
*L*_*d*_: The length of sequencing reads
*S*_*d*_: The sequencing depth
*T*_1_: Threshold for nonrepeats extension
*T*_2_: Threshold for repeats extension.
