# Supplementary material for: A De Novo Genome Assembly Algorithm for Repeats and Nonrepeats
Source: Biomed Res Int. 2014 May 25;2014:736473. doi: 10.1155/2014/736473 (PMC4055594; doi:10.1155/2014/736473)
Supplement: Supplementary file 1 — The supplementary materials include three parts: the first part is the performances of SWA in two species with extremly different CG contents presented in S-Table 1 and S-Table 2. These two species contained completely differnet CG contents. The results indicated that CG contents has little influences on the performances of SWA; the seconde part is the detailed generation process of three real simulated sequences in MATLAB platform; and the final part is the Appendix which contains the copy number information presented in Table 1-Table 6 and S-Table 1. [file 736473.f1.pdf]

Supplementary materials

In this part, we provide the supporting materials of the manuscript of SWA.

The total length of A. dehalogenans 2CP-C (NC_007760) and P. falciparum 3D7 chr14 (NC_004317) are 3291834bp and 5013479bp respectively. The corresponding base counts of them are presented in S-Table 2. These two chromosomes represent the two extreme examples of CG contents. The CG contents of them are 18.4% and 74.9% respectively.

S-Table 1. The results of SWA in datasets A. dehalogenans 2CP-C (NC_007760) and P. falciparum 3D7 chr14 (NC_004317)

Species	Repeat			Contigs.(kb)		Accuracy(%)			Genome
	TRC	CNRC	NNC	N50	Max	CN-accuracy	Rep-accuracy	C-accuracy	coverage(%)
A.deha	50	appendix	1485	4.1	29.1	96%	87%	100%	102.6%
Chr14-P.falc	37	appendix	865	18.7	65.9	95.3%	91.9%	100%	100.2%

Contigs of repeat and non-repeat are generated in independent way by SWA with basic parameters: sequencing depth S_d=2, read length $L_r = 50$, filtered times=1, sliding window=3. Contigs smaller than 80 are removed.

From the S-Table 1, we can clearly see that SWA performs well in these two chromosomes. For A.deha, the CG content is only 18.4%. The performances of SWA are still good, especially for the accuracy of assembled repeats. The metrics such as TRC, CN-accuracy, Rep-accuracy and C-accuracy are very good. For chr14-P.falc, the CG content is only 74.9%. The performances of SWA are still good, especially for the accuracy of assembled repeats. The metrics such as TRC, CN-accuracy, Rep-accuracy and C-accuracy are very good. All of these indicate that the CG contents have little to the performances of assembling repeats and non-repeats independently, which validate that SWA is robust to the CG contents.

S-Table 2. The CG contents of A.deha and chr14-P.falc

base	A. dehalogenans	P. falciparum 3D7 chr14
A	1338715	626051
C	301268	1882373
G	305650	1872987
T	1346201	632068

S-Table 3. The detailed information of repeats contained in simulated datasets

Model sequences	Repetitive contents	Length	Copies
Sequence A	Interspersed repeats	500	8
		1000	10
		2000	8
		3000	6
		4000	5

		5000	3
Sequence B	Tandem repeats	1000 and 5000	5
		2000 and 4000	4
		3000 and 3000	6
Sequence C	Compound repeats	Sequence A and Sequence B	

The following is the detailed generation process of three real simulated datasets, which contain different contents of repeats.

Sequence A contains interspersed repeats

```
SequenceA=randseq(500000);
>> b=randseq(500);
>> c=randseq(1000);
>> d=randseq(2000);
>> e=randseq(3000);
>> f=randseq(4000);
>> g=randseq(5000);
>> SequenceA(1001:1500)=b;
>> SequenceA(2001:2500)=b;
>> SequenceA(3001:3500)=b;
>> SequenceA(4001:4500)=b;
>> SequenceA(5001:5500)=b;
>> SequenceA(6001:6500)=b;
>> SequenceA(8001:8500)=b;
>> SequenceA(9001:9500)=b;
>> SequenceA(12001:13000)=c;
>> SequenceA(22001:23000)=c;
>> SequenceA(32001:33000)=c;
>> SequenceA(42001:43000)=c;
>> SequenceA(52001:53000)=c;
>> SequenceA(62001:63000)=c;
>> SequenceA(72001:73000)=c;
>> SequenceA(82001:83000)=c;
>> SequenceA(92001:93000)=c;
>> SequenceA(102001:103000)=c;
>> SequenceA(202001:204000)=d;
>> SequenceA(252001:254000)=d;
>> SequenceA(272001:274000)=d;
>> SequenceA(292001:294000)=d;
>> SequenceA(322001:324000)=d;
>> SequenceA(342001:344000)=d;
>> SequenceA(362001:364000)=d;
>> SequenceA(382001:384000)=d;
>> SequenceA(402001:405000)=e;
>> SequenceA(422001:425000)=e;
>> SequenceA(442001:445000)=e;
>> SequenceA(452001:455000)=e;
>> SequenceA(462001:465000)=e;
>> SequenceA(466001:469000)=e;
>> SequenceA(472001:476000)=f;
>> SequenceA(477001:481000)=f;
>> SequenceA(483001:487000)=f;
>> SequenceA(488001:492000)=f;
>> SequenceA(493001:497000)=f;
>> SequenceA(260001:265000)=g;
>> SequenceA(280001:285000)=g;
>> SequenceA(300001:305000)=g;
```

Sequence B contains tandem repeats.

```
Sequence B=randseq(500000);
>> b=randseq(500);
>> c=randseq(1000);
>> d=randseq(2000);
>> e=randseq(3000);
>> f=randseq(4000);
>> SequenceB(1001:1500)=b;
>> SequenceB(1501:2000)=b;
>> SequenceB(2001:2500)=b;
>> SequenceB(5001:5500)=b;
>> SequenceB(5501:6000)=b;
>> SequenceB(6001:6500)=b;
```

```
>> SequenceB(8001:8500)=b;
>> SequenceB(8501:9000)=b;
>> SequenceB(9001:9500)=b;
>> SequenceB(10001:11000)=c;
>> SequenceB(11001:12000)=c;
>> SequenceB(12001:13000)=c;
>> SequenceB(13001:14000)=c;
>> SequenceB(14001:15000)=c;
>> SequenceB(24001:25000)=c;
>> SequenceB(25001:26000)=c;
>> SequenceB(26001:27000)=c;
>> SequenceB(27001:28000)=c;
>> SequenceB(28001:29000)=c;
>> SequenceB(41001:42000)=c;
>> SequenceB(42001:43000)=c;
>> SequenceB(43001:44000)=c;
>> SequenceB(44001:45000)=c;
>> SequenceB(45001:46000)=c;
>> SequenceB(65001:66000)=c;
>> SequenceB(66001:67000)=c;
>> SequenceB(67001:68000)=c;
>> SequenceB(68001:69000)=c;
>> SequenceB(69001:70000)=c;
>> SequenceB(101001:103000)=d;
>> SequenceB(103001:105000)=d;
>> SequenceB(105001:107000)=d;
>> SequenceB(201001:203000)=d;
>> SequenceB(203001:205000)=d;
>> SequenceB(205001:207000)=d;
>> SequenceB(251001:253000)=d;
>> SequenceB(253001:255000)=d;
>> SequenceB(255001:257000)=d;
>> SequenceB(303001:305000)=d;
>> SequenceB(305001:307000)=d;
>> SequenceB(307001:309000)=d;
>> SequenceB(323001:325000)=d;
>> SequenceB(325001:327000)=d;
>> SequenceB(327001:329000)=d;
>> SequenceB(345001:347000)=d;
>> SequenceB(347001:349000)=d;
>> SequenceB(349001:351000)=d;
>> SequenceB(361001:364000)=e;
>> SequenceB(364001:367000)=e;
>> SequenceB(367001:370000)=e;
>> SequenceB(372001:375000)=e;
>> SequenceB(375001:378000)=e;
>> SequenceB(378001:381000)=e;
>> SequenceB(383001:386000)=e;
>> SequenceB(386001:389000)=e;
>> SequenceB(389001:392000)=e;
>> SequenceB(393001:397000)=f;
>> SequenceB(397001:401000)=f;
>> SequenceB(403001:407000)=f;
>> SequenceB(407001:411000)=f;
>> SequenceB(427001:421000)=f;
>> SequenceB(427001:431000)=f;
>> SequenceB(431001:435000)=f;
>> SequenceB(440001:445000)=g;
>> SequenceB(445001:450000)=g;
>> SequenceB(475001:480000)=g;
>> SequenceB(480001:485000)=g;
>> SequenceB(490001:495000)=g;
>> SequenceB(495001:500000)=g;
```

Sequence C contains compound repeats

SequenceC=SequenceA+SequenceB

Appendix

Table 1:

Compound repeats—CNRC={[6][6][18][9] [8][3] [4][5][20][10][9][8]}

Table 2:

Sequencing depth=2: CNRC={[3][4][2][6][2][5][2][2][2]}

Sequencing depth=1: CNRC={[3][2][4][6][5][2][2][2][2][2][2][2][2][2][2]}

Sequencing depth=0.5: CNRC={[3][4][3][6][3][3][3][5][3][3][3][2][3][3]}

Sequencing depth=0.2:CNRC={[4][6][5][3][4][5][5][3][3][4][4][4][3][4][4][3][4]}

Table 3:

Read length=36: CNRC={[3][2][4][6][5][2][2][2][2][2]}

Read length=50: CNRC={[3][4][2][6][5][2][2][2][2]}

Table 4:

Window size=3: CNRC={[3][4][3][6][5][3][2][2][2][2][2]}

Window size=7: CNRC={[3][4][2][6][2][5][2][2][2][2]}

Window size=11: CNRC={[3][4][2][6][2][2][5][2][2][2][2][2][2][2][2]}

Table 5:

Filtered times=1: CNRC={[6][4][4][5][3][3][3][3][3][3][2]}

Filtered times=2: CNRC={[4][5][3][7][2][3][6][3][2][3][2][3]}

Filtered times=3: CNRC={[3][4][3][6][4][5][3][3][3][3][2][3][3]}

Table 6:

chrIV-S.c—CNRC={ [3][3][2][2][3][2][2][3][2][2][3][2][2][2][2][2][2][4][2][3][3][2][2][2][3][2][2][2][2][2][4][2]}

E.coli—CNRC={[12][5][10][4][2][3][10][14][2][3][7][10][14][2][2][2][5][5][5][2][2][2][2][2][4][5][4][2][2][15][15][2][9][2][2][2][4][2][2][2][2][2][2][2][2][2][2][2][2][2]}

chrIII-S.e--CNRC={[11][3][13][2][5][2][2][4][3][2][2][2][13][2][3][2][7][2][23][2][2][2][2][4][2][5][4][8][19][2][2][2][2][3][10][2][3][2][7][2][10][3][2][3][7][3][18][2][2][15][22][2][6][3][3][6][2][3][2][3][2][3][2][3][2][2][2][2][3][2][10][2][8][2][2][2][7][2][6][2][2][3][8][2][2][2][2][26][23][2][2][2][2][3][2][2][3][2][2][2][2][2][2][2][2][2][2][3][2][2][2][2][2][2][2][2][6][2][2][2][2][3][3][2][2][2][3][11][6][2][2][2][2][2][5][2][2][2][2][2][2][2][2][2][2][3][3][2][2][3][2][2][5][2][2][2][2][4][2][2][2][2][2][2][2][2][2][2][7][10][2][2][2][9][2][7][3][2][2][2][2][2][2][2][2][2][2][4][2][2][2][2][2][2][2][2][2][2]}

S-Table 1:

A.deha—CNRC={[31][2][2][2][3][2][3][2][61][2][2][2][2][2][2][2][2][2][2][24][4][4][2][3][2][2][2][3][2][2][3][2][26][2][2][4][3][65][58][2][3][4][2][3][3][2][2][2][2][2]}

Chr14-P.falc—CNRC={[4][2][2][2][3][2][2][2][2][2][2][3][3][2][2][2][2][2][2][2][3][2][3][2][2][2][2][3][2][2][3][2][2][2][3][2][3]}
